# Transdermal Semaglutide Administration in Mice: Reduces Body Weight by Suppressing Appetite and Enhancing Metabolic Rate

**DOI:** 10.3390/biology14050575

**Published:** 2025-05-20

**Authors:** Wenjing Li, Ruilin Cai, Binxin Yin, Yingying Zhou, Xinyuan Dong, Wenting Li, Jing Wen

**Affiliations:** 1 College of Life and Environmental Science, Wenzhou University, Wenzhou 325035, China; 23211231128@stu.wzu.edu.cn (W.L.); 23211231210@stu.wzu.edu.cn (R.C.); 23211231125@stu.wzu.edu.cn (B.Y.); 23211231122@stu.wzu.edu.cn (Y.Z.); dongxinyuan@stu.wzu.edu.cn (X.D.); lwt@stu.wzu.edu.cn (W.L.); 2 Zhejiang Provincial Key Laboratory for Water Environment and Marine Biological Resources Protection, Wenzhou University, Wenzhou 325035, China

**Keywords:** GLP-1RA, transdermal, body weight, metabolic rate, anxiety-like behavior

## Abstract

Background: Semaglutide is a glucagon-like peptide-1 (GLP-1) receptor agonist that shows significant efficacy in treating obesity. However, its associated side effects, including poor patient compliance and gastrointestinal inflammation, are concerning and may be largely attributed to its administration methods (e.g., injection vs. oral) and the pronounced fluctuations in systemic drug concentrations. To address these challenges, we investigated an innovative drug delivery system (Transdermal Drug Delivery System, TDDS) designed to maintain therapeutic efficacy while improving patient adherence. Results: Both transdermal and injection treatments of semaglutide decreased body weight, carcass weight, blood glucose, and triglyceride levels in male mice compared with the vehicle-treated control group. In addition, transdermal semaglutide in mice reduced the expression of feeding neuropeptides and the mass of the digestive tract, but increased brown adipose tissue (BAT) mass, metabolic rate, and physical activity, compared with the semaglutide injection group. Additionally, transdermal semaglutide had anxiolytic effects on behavior and did not alter tissue pathology in mice. Conclusion: Compared with the injection paradigm, transdermal semaglutide treatment achieved superior weight loss results in two possible ways: It may reduce energy intake by decreasing the expression of feeding neuropeptides and reducing the weight of the digestive tract. It may also facilitate energy expenditure by enhancing physical activity and increasing BAT mass to boost the metabolic rate. Transdermal semaglutide treatment also has an anxiolytic effect on behavior. Together, our data suggest that TDDS treatment of GLP-1RA may have superior clinical safety and sustainability, providing a novel, efficient, and low-risk obesity treatment.

## Simple Summary

In order to explore the effect of an innovative semaglutide delivery system on obesity, we compared transdermal semaglutide treatment with the injection paradigm. The findings revealed that transdermal semaglutide treatment achieved superior weight loss results, possibly by regulating the expression of feeding neuropeptides to decrease energy intake and by enhancing physical activity to increase energy expenditure. These findings can deepen our understanding of the potential beneficial effects of transdermal drug delivery system (TDDS) treatment on body composition, physiological processes, and behaviors.

## 1. Introduction

Obesity has become a global pandemic, with its prevalence tripling since 1975 [1]. Currently, the global overweight population exceeds 2 billion, accounting for approximately 30% of the world’s population [2]. Obesity significantly impacts human health by contributing to numerous comorbidities, including type 2 diabetes, hypertension, cardiovascular diseases, certain malignancies, and mental health disorders such as depression and anxiety [3,4,5]. It is also associated with reduced life expectancy, diminished quality of life, and an increased burden on healthcare systems [6].

Unfortunately, obesity management is facing an unprecedented challenge. Traditional interventions like dietary restrictions and exercise are central to weight management. However, maintaining these interventions over the long term remains challenging because of their physical and psychological demands on patients [7]. Pharmacological interventions have become increasingly prevalent. Among these, glucagon-like peptide-1 receptor agonists (GLP-1RA), such as liraglutide, exenatide, and semaglutide, have emerged as effective treatments for obesity and its related metabolic decrease. These drugs enhance glucose metabolism, delay gastric emptying, and suppress appetite, leading to a decrease in body weight and cardiovascular disease risk [7]. However, it is crucial to consider the related gastrointestinal side effects, discomfort from injections, and patients’ poor compliance. These factors have significantly impeded the widespread adoption of GLP-1 therapy in clinical settings [8,9]. For example, semaglutide, a second-generation GLP-1 receptor agonist, was approved by the Food and Drug Administration (FDA) in 2021 for the treatment of obesity in adults with a body mass index (BMI) ≥30 kg/m^2^ or ≥27 kg/m^2^ with body weight-related complications [10]. It demonstrates superior efficacy in reducing body weight compared with previous GLP-1 therapies and offers a once-weekly dosing regimen, providing patients with greater convenience [10]. However, its side effects, including gastrointestinal discomfort, pancreatitis, gallbladder disease, and rare risks of thyroid or pancreatic cancer, may hinder long-term adherence [8]. Additionally, most GLP-1 drugs are injectable, which is often poorly tolerated by patients. This highlights the importance of developing alternative delivery methods to boost efficacy and to improve users’ compliance.

To address these limitations, researchers have explored novel drug delivery systems. With technological advancements and evolving human requirements, drug delivery methods have trended toward convenience, transitioning from traditional injections, and oral medications to transdermal patch administration [11,12]. Transdermal patches have attracted considerable attention as a non-invasive and patient-friendly alternative. Transdermal patches offer several advantages: they bypass gastrointestinal metabolism, minimize systemic side effects, maintain stable blood drug concentrations, and reduce the frequency of drug administration [13]. Furthermore, transdermal patches have already proven successful in other therapeutic areas, such as pain management, hormonal replacement therapy, and cardiovascular disease treatment, establishing a precedent for their potential application in obesity management [14]. These properties make transdermal patches an ideal platform for GLP-1 therapy delivery. However, their application in GLP-1RA is restricted by challenges such as the poor skin permeability of macromolecular drugs and the need for advanced formulation techniques [15,16]. Recently, chemical enhancers and nanocarrier systems have improved skin permeability and the multilayer design of modern patches has allowed for the sustained release of active compounds [17], demonstrating significant potential in overcoming the challenges associated with applying transdermal patches to GLP-1RAs.

In this study, we developed a GLP-1 transdermal patch that combines the therapeutic efficacy of GLP-1 receptor agonists with the convenience and improved patient adherence offered by transdermal delivery. Using a high-fat diet-induced obesity model in C57BL/6J male mice, we evaluated the patch’s ability to reduce body weight and to improve metabolic parameters, compared with conventional injection methods. This study addresses current GLP-1 therapy limitations, offering an innovative and effective obesity treatment solution for obesity and its related comorbidities.

## 2. Materials and Methods

### 2.1. Animals and Treatment

Eighteen male C57BL/6J mice (8–12 weeks old) were purchased from Beijing Vital River Laboratory Animal Technology Co., Ltd. (Beijing, China). The mice were housed individually in plastic cages (29 cm × 18 cm × 16 cm) with sawdust bedding and maintained under standard laboratory conditions (23 ± 1 °C, 12:12 h light/dark cycle, lights on at 08:00). All animals had ad libitum access to water and a high-calorie diet (22.0 kJ/g; Xie-tong Pharmaceutical Bioengineering Co., Ltd., Nanjing, China) that consisted of 60% fat, 20% carbohydrates, and 20% protein. Mice were randomly assigned to one of three groups (n = 6 per group): vehicle control group, who received weekly subcutaneous injections of 100 µL phosphate-buffered saline (PBS); positive control group (injection), who received weekly subcutaneous injections of 100 µL PBS with semaglutide (10 µg; Novo Nordisk, Bagsværd, Denmark); and TDDS (Transdermal Drug Delivery System) treatment group, who underwent dorsal hair removal weekly, followed by application of a semaglutide-loaded transdermal patch (35 mm × 15 mm containing 2 mg semaglutide) for 8 h, after which the patch was removed. By the end of the experiment, mice were sacrificed, their blood and organs, including the brain, intestine, lung, liver, spleen, kidney, pancreas, brown adipose tissue (BAT), and heart were collected.

### 2.2. Body Mass

All treatments and measurements were conducted at 7:00 p.m. Animals were weighed daily to the nearest 0.1 g.

### 2.3. Blood Indexes

Serum cholesterol (CHOL), low-density lipoprotein cholesterol (LDL-C), high-density lipoprotein cholesterol (HDL-C), and triglycerides (TG) were assayed using a ROCHE COBAS C702 automated clinical chemistry analyzer (Roche Diagnostics, Indianapolis, IN, USA) with strict quality control procedures. CHOL concentrations were measured using the cholesterol oxidase method, LDL-C and HDL-C concentrations were determined by direct methods, and TG concentrations were measured by an enzymatic method [18]. Blood glucose levels were measured via tail tip blood sampling using glucometer strips (Sinocare, Changsha, China) [19].

### 2.4. Metabolic Indexes

After 7 weeks of treatment, the metabolic rate (MR) and resting metabolic rate (RMR) were measured using previously established methods [20]. MR and RMR were quantified as the rate of oxygen consumption, measured with an O_2_ module high-speed sensor unit (994620-CSHSP-01) for caloric metric measurements in an open-flow respirometry system (TSE, Thuringia, Germany). Air was pumped through a cylindrical sealed Perspex chamber at a rate of 1 L/min. Gases leaving the chamber were dried and sampled using an oxygen analyzer at a flow rate of 0.38 L/min. Data were collected every 10 s by a computer connected via an analogue-to-digital converter (TSE, Thuringia, Germany). MR was measured for 24 h during which food and water were provided ad libitum, lights were turned on between 8:00 and 20:00 at a temperature of 23 ± 0.5 °C. RMR was measured between 10:00 and 17:00 (lights were turned on between 8:00 and 20:00) at a temperature of 30 ± 0.5 °C, which is within the thermal neutral zone of this species. RMR was calculated as the average of the consecutive 10 min minimum oxygen consumption rates, corrected to standard temperature and air pressure conditions, and expressed as mLO_2_/h.

### 2.5. Behavioral Measurements

At the end of the 7-week treatment, anxiety-like behavior was evaluated in animals using the Elevated Plus Maze (EPM) and Open Field (OF) tests, as described in our previous studies [21]. For the EPM test, each animal was gently placed at the center of the EPM apparatus and allowed to explore for 5 min. The EPM apparatus comprised two open arms and two closed arms, each measuring 30 cm × 5 cm, connected at a central platform (5 cm× 5 cm). The closed arms (CA) were enclosed by 17 cm high walls, while the open arms (OA) were wall-free. The entire apparatus was constructed from white plastic, suspended 75 cm above the floor. One day after the EMP test, the OF test was assessed. For the OF test, animals were placed in the center of a 30 cm × 30 cm × 40 cm open field box and allowed to explore for 5 min. After each testing session, the apparatus was cleaned with a 70% ethanol solution to eliminate any olfactory cues and organic waste left by the animals.

### 2.6. Body Fat Deposit and Organs Collection

Animals were euthanized by decapitation. Blood was collected, allowed to stand at room temperature for 2 h, and then stored at 4 °C for an additional 6 h. Subsequently, the blood was centrifuged at 3500 rpm for 15 min to separate the serum. The serum was then stored at −20 °C for further analysis. The brain and liver were carefully excised, immediately frozen in liquid nitrogen, and then stored at −80 °C until further analysis. The abdominal fat, peritesticular fat, brown adipose tissue (BAT), mesenteric fat, subcutaneous fat, and organs were also carefully and rapidly excised. The fresh weight was determined using an electronic balance. Gravimetric measurements were performed using analytical balances (BT25S, Sartorius, Göttingen, Germany) with an accuracy of 0.001 g.

### 2.7. Real-Time RT-qPCR Analysis

The mRNA expression levels of neuropeptide Y (*NPY*), cocaine and amphetamine-regulated transcript (*CART*), proopiomelanocortin (*POMC*), agouti-related protein (*AgRP*), and obesity receptor B (*ObRb*) in the hypothalamus were quantified using real-time RT-qPCR analysis. Total RNA was extracted from the hypothalamus using TRIzol Reagent (TAKARA, Dalian, China). cDNA was synthesized in a final reaction volume of 50 μL with random primer oligo (dT)18 and AMV Reverse Transcriptase (TAKARA). Two μL cDNA samples were taken for the subsequent PCR reaction using gene-specific primers (Table 1). The final reaction volume of 20 μL contained 10 μL of 2 × SYBR Premix EX Tag TM (TAKARA), 0.4 μL of forward and reverse primers (final concentration 0.2 μM per primer), 2 μL cDNA template, and 7.2 μL DEPC H_2_O. qPCR was performed using Roche Light Cycler480II real-time qPCR system (Roche Diagnostics, Indianapolis, IN, USA). *Actin* was used as an internal control. Relative gene expression levels were quantified using the comparative cycle threshold (ΔΔCt) method [22].

## 3. Data Analysis

All the data were statistically analyzed and graphed using GraphPad Prism statistical software (version 8.0). One-way analysis of variance (ANOVA) was used to analyze intergroup differences in organ weights, four blood lipid parameters, expression of feeding neuropeptides in the hypothalamus, and behavioral indices. A one-way ANOVA was performed on resting metabolic rate, average daytime and nighttime metabolic rates, with body weight as a covariate. The continuous changes in body weight and blood glucose levels were examined using repeated measures one-way analysis of variance (ANOVA), followed by Student–Newman–Keuls (SNK) post-hoc tests when the main effects were significant. Data were presented as the mean ± standard error of the mean (SEM), and *p* < 0.05 was considered a statistically significant difference.

## 4. Results

### 4.1. Body Weight and Fat Distribution

**Figure 1:**
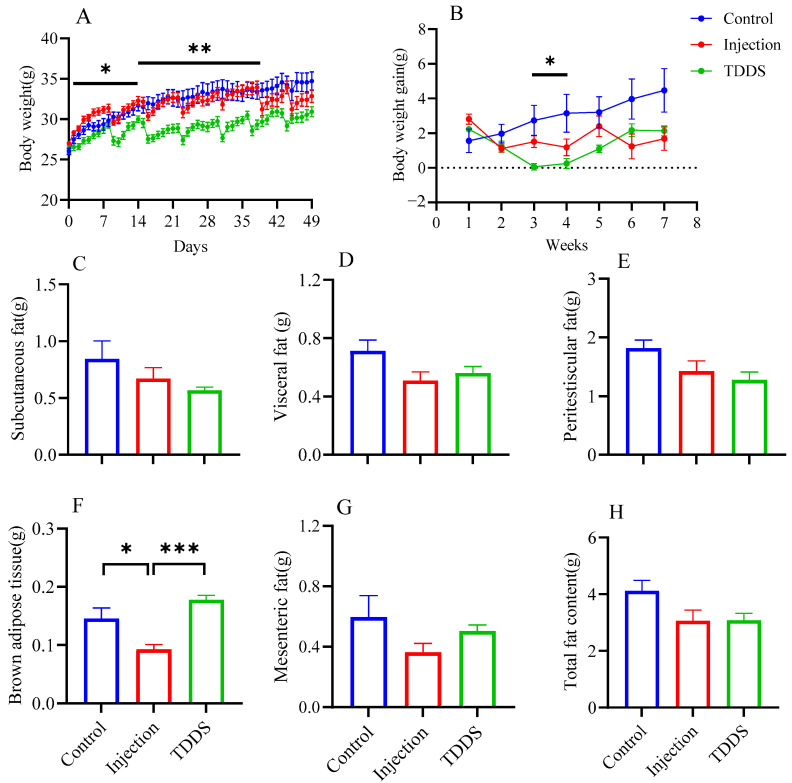
The effects of semaglutide treatments (injection vs. TDDS) on body weight and fat distribution in C57BL/6J male mice. Semaglutide transdermal patches significantly affected body weight (**A**), body weight gain (**B**), and brown adipose tissue (**F**). However, no significant treatment effects were observed on subcutaneous fat (**C**), visceral fat (**D**), peritesticular fat (**E**), mesenteric fat (**G**), and total fat content (**H**). The data are expressed as mean ± SEM, *, *p* < 0.05; **, *p* < 0.01; ***, *p* < 0.001.

Body weight increased progressively in all groups during the high-fat diet feeding period. However, the mode of semaglutide administration significantly affected the trajectory and magnitude of weight gain. Over the 7-week treatment period, body weight in both the injection and TDDS groups continued to increase in a stepwise manner but at significantly slower rates compared with the control group (Figure 1A). The control group exhibited the highest average weekly weight gain (3.83 g/week, Figure 1B), followed by the injection group (1.52 g/week, Figure 1B) and the TDDS group (1.15 g/week, Figure 1B). Although both groups treated with semaglutide tended to show decreased levels on subcutaneous fat (*F*_2,14_ = 1.87, *p* > 0.05, Figure 1C), visceral fat (*F*_2,14_ = 3.05, *p* > 0.05, Figure 1D), peritesticular fat (*F*_2,14_ = 3.19, *p* > 0.05, Figure 1E), mesenteric fat (*F*_2,14_ = 1.93, *p* > 0.05, Figure 1G), and total fat content (*F*_2,14_ = 3.09, *p* > 0.05, Figure 1H) compared with the control group, no significant group differences were found. Only brown adipose tissue (BAT) mass was significantly affected by treatment (*F*_2,14_ = 14.81, *p* < 0.01, Figure 1F). The injection group was significantly lower than both control and TDDS groups, while no differences were found between the control and TDDS groups.

### 4.2. Blood Indexes

**Figure 2:**
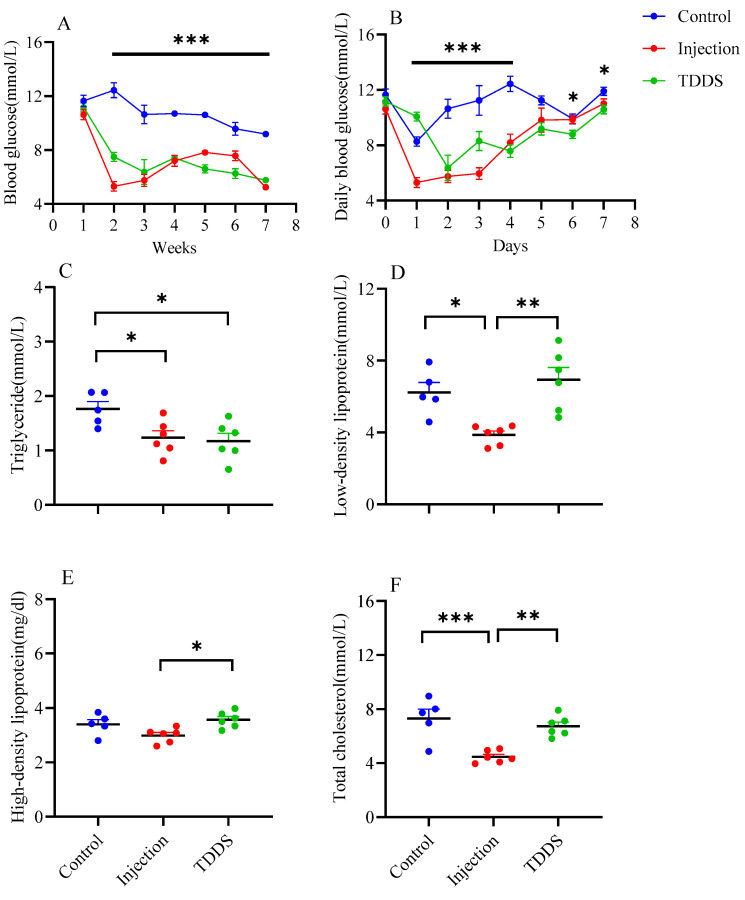
The effect of semaglutide treatments (injection vs. TDDS) on blood indexes in C57BL/6J male mice. Semaglutide treatment significantly affected weekly blood glucose (**A**), daily blood glucose (**B**), and triglyceride (**C**). In addition, the injection group showed lower levels of low-density lipoprotein (**D**), high-density lipoprotein (**E**) and total cholesterol (**F**) than the TDDS group. The data are expressed as mean ± SEM, *, *p* < 0.05; **, *p* < 0.01; ***, *p* < 0.001.

Semaglutide administration significantly influenced blood glucose dynamics in mice. During the experiment, blood glucose levels differed markedly among the three groups (*F*_2,14_ = 25.59, *p* < 0.0001, Figure 2A). Compared with the control group, the first semaglutide administration led to a significant decrease in blood glucose in both the injection and TDDS groups, with reductions of 5.31 mmol/L and 3.67 mmol/L, respectively. To further evaluate short-term glycemic responses, daily blood glucose levels were monitored for seven consecutive days after the first treatment (*F*_14,62_ = 9.85, *p* < 0.0001, Figure 2B). Compared with the control group, the blood glucose of semaglutide-treated mice was lower. The injection group and TDDS group reached the lowest blood glucose levels on day 1 (5.3 mmol/L) and day 2 (6.36 mmol/L) after treatment, respectively. By day 5 after treatment, no group differences were found. Serum triglyceride levels were lower in both the injection and TDDS groups compared with the control group, with no significant difference between the two treatment groups (*F*_2,14_ = 5.36, *p* < 0.05, Figure 2C). The injection group showed significant decreases in low density lipoprotein (*F*_2,14_ = 9.98, *p* < 0.001, Figure 2D) and total cholesterol (*F*_2,14_ = 13.51, *p* < 0.001, Figure 2F), compared with the control and TDDS groups whereas no differences were found between the control and TDDS groups. In addition, the injection group showed a significantly lower level of high-density lipoprotein than the TDDS group (*F*_2,14_ = 5.35, *p* < 0.05, Figure 2E).

### 4.3. Metabolic Rate

**Figure 3:**
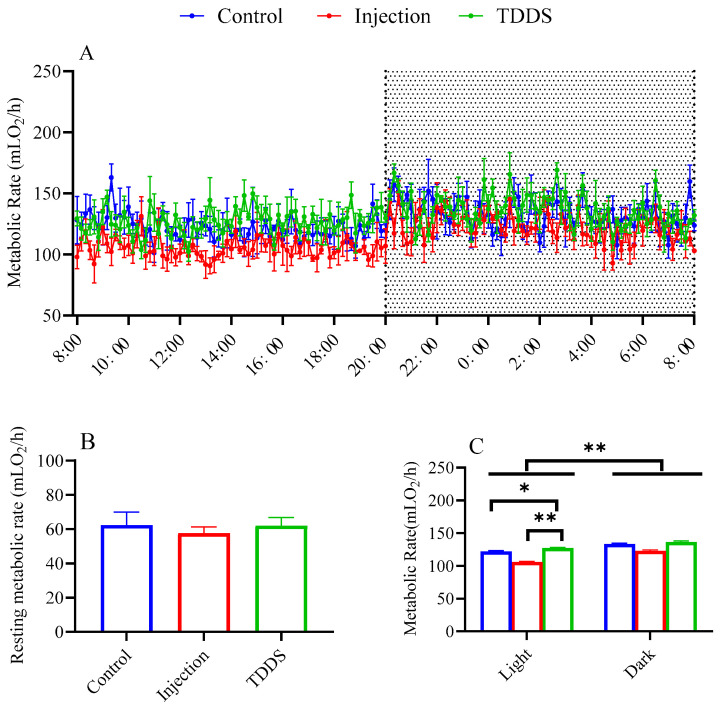
The effect of semaglutide treatments (injection vs. TDDS) on metabolic rate in C57BL/6J male mice. Semaglutide treatment significantly affected metabolic rate (**A**,**C**). However, no significant treatment effects were observed on resting metabolic rate (**B**). The data are expressed as mean ± SEM, *, *p* < 0.05; **, *p* < 0.001.

All three groups exhibited significant circadian rhythms in daily metabolic rate, with nighttime values higher than daytime values (*F*_1,17_ = 9.06, *p* < 0.01, Figure 3A,C). Although no significant group difference was found for resting metabolic rate (*F*_2,8_ = 0.19, *p* > 0.05, Figure 3B), the TDDS group had a significantly higher metabolic rate than the injection group which, in turn, did not differ from the control group (*F*_2,17_ = 8.24, *p* < 0.01, Figure 3C). Similar effects were found in light phases (*F*_2,8_ = 7.61, *p* < 0.05, Figure 3C).

### 4.4. Organ Weight

**Figure 4:**
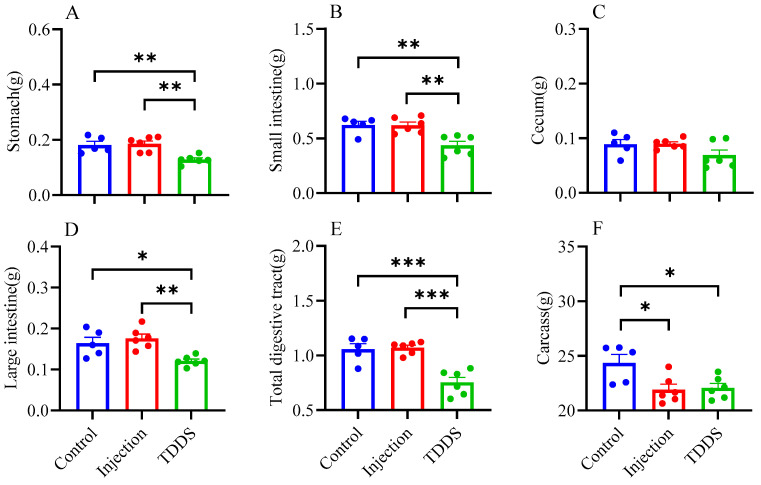
The effect of semaglutide treatments (injection vs. TDDS) on organ weight in C57BL/6J male mice. Semaglutide transdermal patches significantly affected the weight of stomach (**A**), small intestine (**B**), large intestine (**D**), total digestive tract (**E**), and carcass (**F**). However, no significant treatment effects were observed on the cecum (**C**). The data are expressed as mean ± SEM, *, *p* < 0.05; **, *p* < 0.01; ***, *p* < 0.001.

The TDDS group had significantly lower weights for the stomach (*F*_2,14_ = 10.49, *p* < 0.01, Figure 4A), small intestine (*F*_2,14_ = 10.44, *p* < 0.01, Figure 4B), large intestine (*F*_2,14_ = 8.58, *p* < 0.01, Figure 4D), and total digestive tract (*F*_2,14_ = 19.82, *p* < 0.001, Figure 4E) compared with the control and injection groups, which did not differ from each other. In addition, the injection and TDDS groups had lower carcass weights compared with the control (*F*_2,14_ = 5.68, *p* < 0.05, Figure 4F), whereas no group difference was found for the cecum (*F*_2,14_ = 2.39, *p* > 0.05, Figure 4C). The TDDS and control groups had higher liver weights than the injection group, with no significant difference between the former two groups (Table 2). Furthermore, the TDDS group had the highest lung mass, followed by the injection group, while the control group had the lowest (Table 2).

### 4.5. Expression of Feeding-Related Neuropeptides

**Figure 5:**
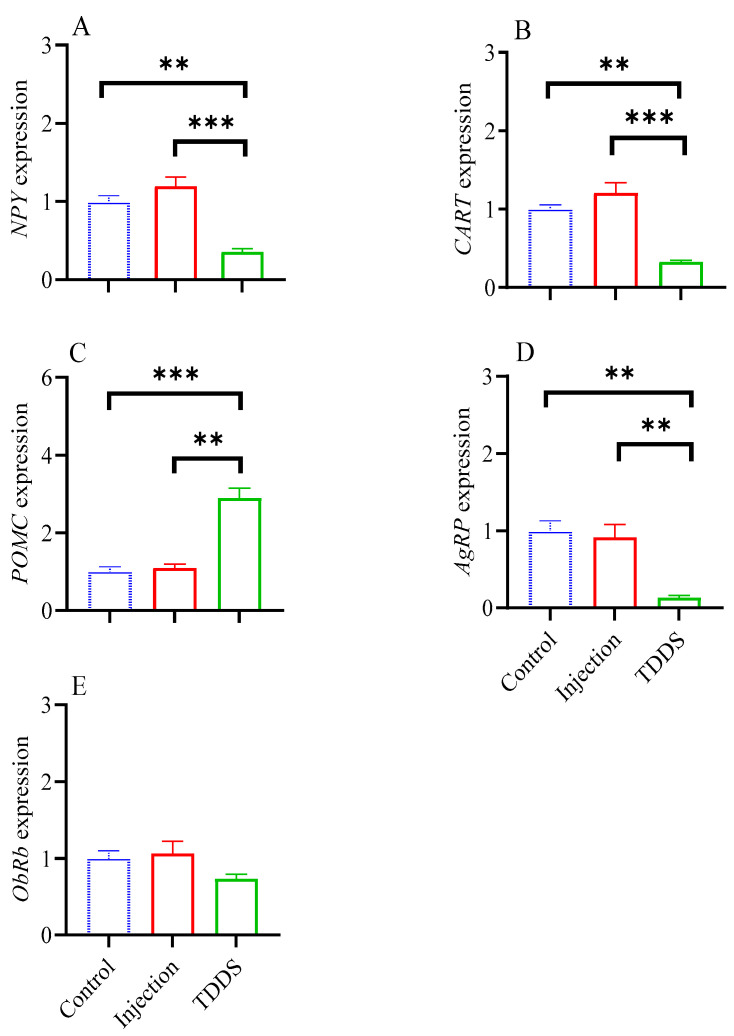
The effect of transdermal semaglutide treatments on feeding-related neuropeptides in C57BL/6J male mice hypothalamus. Transdermal, but not injection, semaglutide treatment significantly affected the gene expression of *NPY* (**A**), *CART* (**B**), POMC (**C**), and *AgRP* (**D**). No group differences were found on *ObRb* expression (**E**). The data are expressed as mean ± SEM, **, *p* < 0.01; ***, *p* < 0.001.

The TDDS group had lower levels of *NPY* (*F*_2,13_ = 15.86, *p* < 0.001, Figure 5A), *CART* (*F*_2,13_ = 17.22, *p* < 0.001, Figure 5B), and *AgRP* (*F*_2,13_ = 11.01, *p* < 0.001, Figure 5D), but a higher level of *POMC* (*F*_2,13_ = 15.59, *p* < 0.001, Figure 5C) compared with the control and injection groups. No group difference was found in *ObRb* (*F*_2,13_ = 1.42, *p* > 0.05, Figure 5E).

### 4.6. Anxiety-like and Exploratory Behaviors

**Figure 6:**
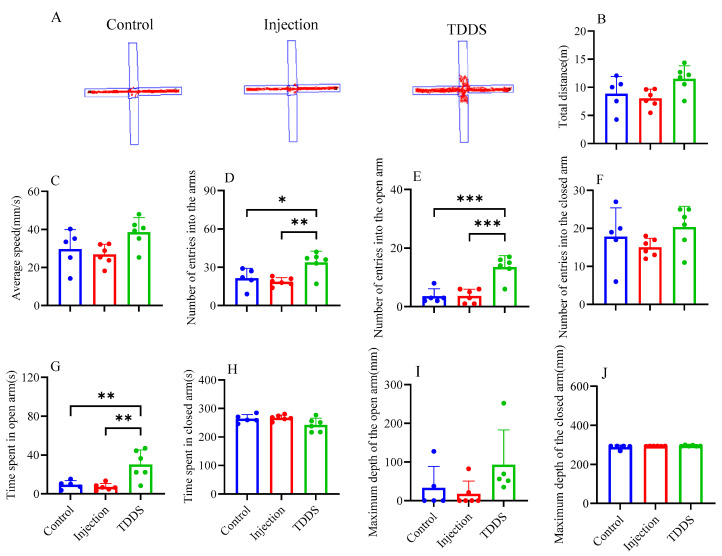
The effect of transdermal semaglutide treatments on the elevated cross maze test in C57BL/6J male mice(**A**). Semaglutide transdermal patches significantly affected the number of entries into the arms (**D**), number of entries into the open arms (**E**), and time spent in the open arms (**G**). No group differences were found in other behavioral measurements: total distance (**B**), average speed (**C**), number of entries into the closed arms (**F**), time spent in closed arms (**H**), maximum depth of entry into the Open Arms (**I**), and maximum depth of entry into the closed arms (**J**). The data are expressed as mean ± SEM, *, *p* < 0.05; **, *p* < 0.01; ***, *p* < 0.001.

In the EPM test, the TDDS group showed a higher number of entries into the arms (*F*_2,14_ = 7.99, *p* < 0.01, Figure 6D), a higher number of entries into the open arms (*F*_2,14_ = 20.33, *p* < 0.001, Figure 6E), and greater time spent in the open arms (*F*_2,14_ = 10.44, *p* < 0.01, Figure 6G) compared with the control and injection groups, which did not differ from each other. No group difference was found in the total distance (*F*_2,14_ = 3.59, *p* > 0.05, Figure 6B), average velocity (*F*_2,14_ = 3.59, *p* > 0.05, Figure 6C), number of entries into the closed arm (*F*_2,14_ = 1.47, *p* > 0.05, Figure 6F), time spent in the closed arm (*F*_2,14_ = 3.64, *p* > 0.05, Figure 6H), maximum depth of the open arm (*F*_2,14_ = 2.18, *p* > 0.05, Figure 6I), and maximum depth of the closed arm (*F*_2,14_ = 1.66, *p* > 0.05, Figure 6J).

**Figure 7:**
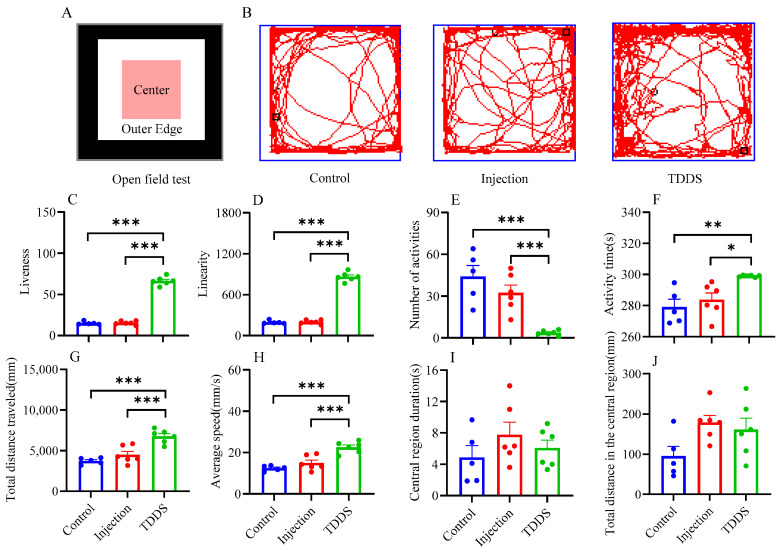
The effects of transdermal semaglutide treatment on the open field test (**A**) in C57BL/6J male mice(**B**). Transdermal semaglutide significantly affected the liveness (**C**), linearity (**D**), number of activities (**E**), activity time (**F**), total distance traveled (**G**), and average speed (**H**) compared with the control and injection groups. No group differences were found for other behavioral measurements: central region duration (**I**) and total distance in the central region (**J**). The data are expressed as mean ± SEM, *, *p* < 0.05; **, *p* < 0.01; ***, *p* < 0.001.

In the OF test, the TDDS group significantly increased mouse liveness (*F*_2,14_ = 419.80, *p* < 0.001, Figure 7C), linearity (*F*_2,14_ = 419.80, *p* < 0.001, Figure 7D), activity time (*F*_2,14_ = 8.14, *p* < 0.01, Figure 7F), total distance traveled (*F*_2,14_ = 20.27, *p* < 0.001 Figure 7G), and average speed (*F*_2,14_ = 20.29, *p* < 0.001, Figure 7H), but decreased the number of activities (*F*_2,14_ = 15.28, *p* < 0.001, Figure 7E) compared with the injection and control groups, which did not differ from each other. Additionally, no group difference was found in central region duration (*F*_2,14_ = 1.07, *p* > 0.05, Figure 7I) and total distance in the central region (*F*_2,14_ = 3.14, *p* > 0.05, Figure 7J).

### 4.7. Hematoxylin-Eosin Staining Pathological Section

**Figure 8:**
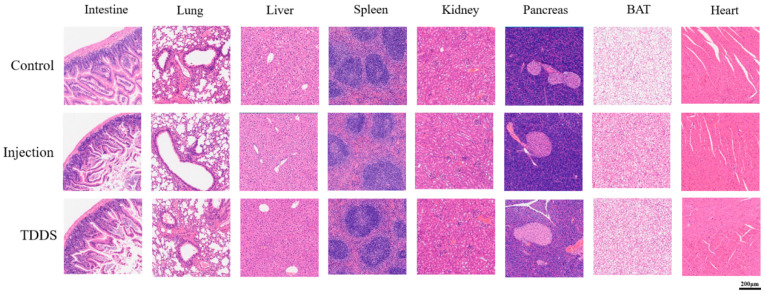
Representative photo illustrations of hematoxylin-eosin staining pathological sections in C57BL/6J male mice. Semaglutide treatment did not cause significant pathological changes in the small intestine, lung, liver, spleen, kidney, pancreas, BAT, and heart under ×40 magnification.

The major organs were collected for hematoxylin and eosin (H&E) staining and histological analysis (Figure 8). After seven consecutive weeks of drug administration, pathological analysis of tissue sections from various organs of mice in each group revealed that changes in the mode of drug administration did not cause significant abnormalities in the lungs, liver, kidneys, and heart. These tissues were within normal histological limits, with no significant signs of inflammation, necrosis, or fibrosis. The lung tissue retained intact alveolar spaces with thin alveolar walls and minimal interstitial space, showing no evidence of inflammatory cell infiltration, edema, fibrosis, or structural abnormalities. The spleen exhibited typical architecture with well-defined white and red pulp. In the kidney sections, the glomeruli and tubules were well preserved, with no indications of glomerular sclerosis, tubular necrosis, or interstitial inflammation. The tubules retained normal morphology, and the renal parenchyma appeared healthy. The cardiac muscle fibers in the myocardium were well organized, exhibiting distinct striations and centrally located nuclei, with no evidence of inflammation, necrosis, fibrosis, hypertrophy, or dilation. Overall, all three groups exhibited no significant toxicity with long-term treatment. However, histological examination revealed notable abnormalities in the small intestine, liver, pancreas, and BAT due to changes in the drug administration mode. In the control group, the intestinal mucosa remained intact, with well-defined villi and crypts. The lamina propria appeared normal, without inflammatory cell infiltration, and the muscularis mucosae were preserved. In contrast, the TDDS and injection groups showed looser intestinal structure, with a significantly thinner muscular layer and shorter, fragmented intestinal epithelial villi compared with the control group. The liver sections of the injection and TDDS groups showed no obvious white granules, which were absent in the control group. Compared with the injection and control groups, the pancreatic sections of the TDDS group exhibited larger acinar lumens. The islet structure remained intact and functional, with no signs of pancreatitis, fibrosis, or endocrine dysfunction. Histological analysis of BAT showed that the brown adipocytes appeared normal, with no signs of inflammation, fibrosis, or necrosis. In the injection and TDDS groups, the brown adipocytes displayed a visibly deeper brown coloration compared with the control group, characterized by multilocular adipocytes. In the control group, the lipid droplets within the adipocytes were significantly larger compared with those in the TDDS and injection groups.

## 5. Discussion

Previous studies indicate that many synthetic GLP-1 receptor agonists exhibit short half-lives, ranging from 2 h to several days, and, thus, frequent dosing is needed to maintain their therapeutic effects [23]. Drug administration methods significantly influence therapeutic efficacy, since different delivery systems affect drug absorption, distribution, and metabolism, thereby exerting distinct impacts on energy metabolism, organ function, and behavioral performance. In the present study, we compared the effects of TDDS and the injectable administration of semaglutide on body weight, fat distribution, metabolic rate, organ function, and behaviors in C57BL/6J male mice. Our data show that, compared with the injection treatment, TDDS treatment achieves better weight loss effects by suppressing appetite and increasing overall activity levels and metabolic rate, without causing pathological changes in the tissue. In addition, TDDS treatment shows anxiolytic effects on behavior.

### 5.1. Transdermal Semaglutide Delivery Is as Effective as Injection Treatment for Reducing Body Weights

Body weight serves as one of the effective criteria for evaluating weight loss efficacy [24]. Our data show that both the TDDS group and the injection group exhibited consistently lower body weight and carcass weight compared with the PBS control group, indicating that both forms of semaglutide treatment can achieve significant weight loss effects. The weight loss may be attributed to changes in body fat mass. Consistent with previous studies, semaglutide-treated mice also showed reduced fat contents in various body regions, such as subcutaneous fat and peritesticular fat [25]. Changes in body fat were also related to the changes in some serum indicators (blood glucose, triglycerides, LDL, HDL, and total cholesterol), which are clinically used to assess glucose lipid metabolism [26]. Both administration methods of semaglutide led to reduced blood glucose and triglyceride levels, implicating that semaglutide treatment can act on increasing glycolysis and lipolysis in the body triggered by insufficient energy intake [27]. The reduction in serum glucose levels with GLP-1RA may also suggest increased insulin sensitivity, consistent with the findings from studies in humans [28]. Additionally, neither administration methods of semaglutide caused significant pathological changes, including inflammation, necrosis, or fibrosis.

### 5.2. Transdermal Semaglutide Delivery Is More Potent than the Injection Treatment

Our data show that GLP-1RA transdermal administration exhibits superior weight loss efficacy compared with the injection method. Meanwhile, the weights of the gastrointestinal tissues in TDDS group were significantly lower than those in the injection group. Previous research has demonstrated that the digestive systems of small mammals, including mice, prairie voles (*Microtus ochrogaster*), and striped hamsters (*Cricetulus barabensis*), exhibit remarkable plasticity, adhering to the principle of matching function to demand [29,30]. A reduction in gut weight may be associated with delayed gastric emptying, diminished digestive and absorptive efficiency, and a decline in food intake [31]. Therefore, we speculate that GLP-1RA transdermal administration may suppress energy intake and nutrient absorption, ultimately leading to greater weight loss in the TDDS group. Further analysis revealed that the levels of the neuropeptides *AgRP* and *NPY*, which stimulate feeding, were significantly decreased in the TDDS group, compared with the injection group. Conversely, the level of the neuropeptide *POMC*, which suppresses feeding, was significantly increased in the TDDS group, indicating that GLP-1 receptor neurons in the dorsomedial hypothalamus may serve as candidates for encoding preingestive satiation in mice, and GLP-1RAs control preingestive satiation in this way [32]. The pronounced difference in hypothalamic neuropeptides expression according to drug delivery methods may stem from TDDS providing a more stable and continuous drug release, which in turn maintains a relatively constant hypothalamic drug concentration and enhances regulation. In contrast, the injection group may have experienced higher peak concentrations followed by a rapid decline, resulting in the less consistent regulation of neuropeptide expression. However, the specific mechanisms of how different drug delivery methods affect the expression of hypothalamic neuropeptides require further research.

The TDDS group exhibited a significantly higher daily metabolic rate than the injection group. This difference may be attributed to enhanced thermogenic capacity or higher activity levels in the TDDS group. Mechanistically, brown adipose tissue (BAT), which plays a crucial role in non-shivering thermogenesis [33], was found to be heavier in the TDDS group, thereby contributing to sustained higher daily metabolic rates through enhanced thermogenesis. TDDS may also promote the browning of white adipose tissue (WAT) [34]. Behavioral analysis revealed that TDDS mice performed with more than 40% higher mobility in the open field tests than the injection group, further supporting the link between activity-driven thermogenesis and systemic energy expenditure [35]. These findings suggest that transdermal GLP-1RA treatment enhances metabolism through two distinct pathways: (1) directly increasing the weight of thermogenic organs and (2) stimulating locomotor activity.

Conversely, the injection treatment appeared to have greater effects in lowering lipoprotein and total cholesterol. However, lower lipoprotein and total cholesterol levels are not always desirable. Previous studies have found that patients with severe liver dysfunction often experience significant reductions in plasma lipoprotein and total cholesterol levels, indicating that long-term injections of semaglutide may pose a risk of liver dysfunction [36]. Daily blood glucose fluctuations revealed that the TDDS group maintained drug efficacy for 3–4 days longer than the injection group, indicating the prolonged action of transdermal semaglutide. This could be attributed to the sustained release property of transdermal GLP-1RA delivery [37].

### 5.3. Transdermal Drug Delivery Has Good Security in Obesity Treatment

In the elevated plus maze and open field tests, TDDS mice displayed increased entry to and time spent in the open arms and the center area, respectively, compared with the injection group. These data indicate that TDDS treatment also has anxiolytic effects on behavior. Although the underlying mechanism is still unknown, TDDS treatment with semaglutide appears to be more beneficial for the brain, mood, and behavior, compared with the injection treatment [38]. It has been shown that transdermal systems, such as Rotigotine, can maintain stable drug concentrations through continuous release, thereby avoiding behavioral issues caused by fluctuations in dopamine receptor agonists [39]. In contrast, injection administration’s rapid onset and concentration fluctuations may differentially affect behavioral patterns [40]. Data from previous studies have shown significant associations between GLP-1RA treatment and increased risks of psychiatric disorders. Specifically, patients on GLP-1RAs exhibited higher risks of major depression, anxiety, and suicidal behavior [41].

## 6. Conclusions

Our data showed that semaglutide treatment was effective in reducing body weight. However, compared with the injection paradigm, transdermal semaglutide treatment achieved superior weight loss results in two possible ways: It may reduce energy intake by regulating the expression of feeding neuropeptides and reducing the weight of the digestive tract. It may also facilitate energy expenditure by enhancing physical activity levels and increasing BAT mass to boost the metabolic rate. Interestingly, TDDS treatment of semaglutide was also found to be anxiolytic, compared with the injection treatment, implicating the potential beneficial effects of TDDS drug treatment on the brain and behaviors. Together, our data suggest that TDDS treatment of GLP-1RA may have superior clinical safety and sustainability, providing a novel, efficient, and low-risk obesity treatment strategy.

## Figures and Tables

**Table 1:** Gene-specific primer sequences used for RT-qPCR analysis.

Gene	Sequence 5′-3′	Size (bp)
*NPY*-f	TCGTGTGTTTGGGCATTCTG	128
*NPY*-r	TCTGGTGATGAGATTGATGTAGTG	
*CART*-f	ACGAGAAGGAGCTGCCAAG	153
*CART*-r	GCTCTCCAGCGTCACACAT	
*POMC*-f	GAAGATGCCGAGATTCTGCT	175
*POMC*-r	CTCCAGCGAGAGGTCGAGTT	
*AgRP*-f	ACCTTAGGGAGGCACCTCAT	151
*AgRP*-r	AGCAACATTGCAGTCAGCAT	
*ObRb*-f	TAAAGCTCTCGTGGCGCTCT	195
*ObRb*-r	TCCACACGAGCAAGAACAAC	
*β-Actin*-f	CGTAAAGACCTCTATGCCAA	317
*β-Actin*-r	GCGCAAGTTAGGTTTTGTC	

**Table 2:** The effect of semaglutide treatments (injection vs. TDDS) on visceral organ weight in C57BL/6J male mice.

Organs	Control	Injection	TDDS	*p*
Heart	0.169 ± 0.014	0.148 ± 0.006	0.1712 ± 0.008	ns
Liver	1.236 ± 0.036 ^b^	1.0942 ± 0.018 ^a^	1.201 ± 0.042 ^b^	*
Spleen	0.072 ± 0.006	0.062 ± 0.003	0.062 ± 0.003	ns
Lung	0.149 ± 0.009 ^a^	0.161 ± 0.007 ^b^	0.182 ± 0.007 ^c^	*
Kidney	0.343 ± 0.012	0.329 ± 0.011	0.312 ± 0.006	ns

The data are expressed as mean ± SEM, ns, *p* > 0.05; *, *p* < 0.05. Different letters indicate significant differences between groups.

## Data Availability

The original contributions presented in this study are included in the article. Further inquiries can be directed to the corresponding author.
